# Exploring the Beliefs, Perceptions, and Experiences of Individuals With Tendinopathy: A Systematic Review and Meta-Ethnography of Qualitative Studies

**DOI:** 10.1093/ptj/pzaf060

**Published:** 2025-04-28

**Authors:** Mark S Mesiha, Steven J Obst, Samantha Randall, Amanda L Rebar, Cassandra K Dittman, Luke J Heales

**Affiliations:** Musculoskeletal Health and Rehabilitations Research Group, School of Health, Medical and Applied Sciences, College of Health and Exercise Science, Central Queensland University, Sydney, NSW 2000, Australia; Musculoskeletal Health and Rehabilitations Research Group, School of Health, Medical and Applied Sciences, College of Health and Exercise Science, Central Queensland University, Bundaberg, QLD 4670, Australia; Musculoskeletal Health and Rehabilitations Research Group, School of Health, Medical and Applied Sciences, College of Health and Exercise Science, Central Queensland University, Rockhampton, QLD 4701, Australia; Motivation of Health Behaviours Lab, Appleton Institute, School of Health, Medical and Applied Sciences, Central Queensland University, Rockhampton, QLD 4701, Australia; Cluster for Resilience and Wellbeing, Appleton Institute, School of Health, Medical and Applied Sciences, Central Queensland University, Bundaberg, QLD 4670, Australia; Manna Institute, Central Queensland University, Bundaberg, QLD 4670, Australia; Musculoskeletal Health and Rehabilitations Research Group, School of Health, Medical and Applied Sciences, College of Health and Exercise Science, Central Queensland University, Rockhampton, QLD 4701, Australia

**Keywords:** Belief, Experience, Meta-ethnography, Perception, Qualitative Research, Tendinosis, Tendinitis, Tendinopathy

## Abstract

**Importance:**

This study systematically examines the effects of tendinopathy on patients’ quality of life and investigates their experiences with rehabilitation.

**Objective:**

This study aimed to synthesize qualitative research exploring the beliefs, perceptions, and experiences of individuals living with tendinopathy by employing a systematic review with meta-ethnography.

**Data Sources:**

Studies were identified from 4 databases (CINAHL, EMBASE, Scopus, and ProQuest One Academic).

**Study Selection:**

Studies were included if they utilized qualitative methods to investigate beliefs, perceptions, and/or experiences of participants with clinically diagnosed tendinopathy.

**Data Extraction and Synthesis:**

Data synthesis was completed using the 7 phases of meta-ethnography and reported using the meta-ethnography reporting guidelines. Risk of bias was assessed using the Joanna Briggs Checklist for Qualitative Studies. Confidence in the findings was assessed using the Grading of Recommendations Assessment, Development, and Evaluation Confidence in the Evidence from Reviews of Qualitative research (GRADE-CERQual).

**Main Outcomes(s) and Measure(s):**

Twenty-three studies were included (rotator cuff [*n* = 12]; Achilles [*n* = 6]; gluteal [*n* = 2]; lateral elbow [*n* = 2]; and mixed tendinopathies [*n* = 1]). Methodological quality of included studies varied. Moderate confidence in review findings 1 and 2 and high confidence in review finding 3.

**Results:**

Qualitative synthesis identified 3 themes: (1) I need to understand why my tendon hurts (participants wanted clarity regarding the cause of symptoms); (2) I want to fix my tendon, but I don’t know how (participants had varied beliefs regarding optimal management and how to reduce their pain); and (3) I am uncertain whether my lifestyle will return to normal (participants felt frustrated with the negative impact that tendinopathy had on their life).

**Conclusion and Relevance:**

This review provides insights into the lived experiences of individuals with tendinopathy. The review advocates for clearer communication and education regarding causes and optimal management of tendinopathy. Participants’ varied beliefs and uncertainties about treatment efficacy suggest that health care providers consider individualized evidence-based guidance to improve patient outcomes.

## INTRODUCTION

Tendinopathy describes persistent activity-related tendon pain.^1^ Despite a relatively simple clinical diagnosis, symptoms of tendinopathy are difficult to manage and recovery can take between 6 and 12 months.^2^ Evidence from patient-reported outcome measures in tendinopathy shows that persistent tendon pain reduces patients’ involvement in recreational, social, and occupational activities.^3^ However, patient-reported outcome measures are often quantitative and lack the ability to explore patients’ experiences of the assessment, diagnosis, and treatment process. Through exploring individuals’ experiences living with tendinopathy, qualitative research may better understand internal/covert (ie, beliefs and cognitions about their injury) and external/overt factors (ie, environmental or occupational impacts) that influence patient progress. Understanding patients’ lived experience of tendinopathy may enable clinicians to develop comprehensive strategies to improve patient outcomes.

Research has explored patients’ experiences living with tendinopathy including the Achilles tendon,^4^ rotator cuff,^5^ and lateral elbow,^6^^,^^7^ identifying variability in patients’ experiences even with the same location of pathology. For instance, in 1 qualitative study examining experiences of 15 individuals with Achilles tendinopathy, some participants were concerned about their ability to return to usual activity levels, while others were optimistic about their recovery.^8^ Another qualitative study on rotator cuff tendinopathy (*n* = 10) revealed that some participants feared “permanent damage” from physical activity, while others emphasized recovery through specific, task-based movements.^9^ Taken together, it appears that patients with persistent tendon pain, even of the same anatomical region, report a range of experiences, which prevents a “1-size-fits-all” approach to conservative management. This variability of qualitative study findings is consistent with the complex and individualized nature of persistent pain, which often encompasses psychological (eg, fear avoidance^10^) and social factors (eg, loss of leisure time^3^). A high-quality comprehensive synthesis of the literature may yield valuable insights into the multifaceted management of these patients, acknowledging that similar anatomical presentations of tendinopathy may not necessarily translate to comparable patient experiences. Therefore, this review aimed to systematically identify and synthesize qualitative studies examining patient’s beliefs, perceptions, and experiences of living with and managing tendinopathy. This systematic review was necessary to (1) understand individual variation in experiences living with tendinopathy, even when the affected tendon is in the same anatomical location, (2) identify common themes in patient experiences that may not be apparent from individual studies alone, and (3) inform health care professionals about the diverse needs of individuals with tendinopathy.

## METHODS

### Design

This systematic review and meta-ethnography followed the Preferred Reporting Items for Systematic Reviews and Meta-Analyses (PRISMA) statement^11^ and the meta-ethnography reporting guidelines (eMERGe).^12^^,^^13^ For more information about the eMERGe, refer to Supplementary Material 1. Meta-ethnography was chosen for its suitability for the synthesizing different types of qualitative research (eg, phenomenology and ethnographic)^12–15^ and its application across health care and social science.^14^^,^^15^ Unlike other synthesis methods, meta-ethnography aims to generate new interpretations and conceptual models rather than merely aggregating findings.^15^^,^^16^ The translation of research findings involved both reciprocal translation, in which similarities between concepts across studies were identified; and refutational translation, in which contradictions between concepts in different studies were identified and explored. The protocol was prospectively registered with the International Prospective Register of Systematic Reviews (PROSPERO; CRD42022313718).

### Data Sources and Searches

CINAHL, EMBASE, and Scopus were searched in August 2023 using key words: (experience OR belief OR perception) AND (tendinosis OR tendinitis OR tendinopathy OR tendinopathy OR epicondylalgia OR epicondylitis OR jumpers knee OR tennis elbow OR golfers elbow OR gluteus OR patella OR rotator cuff). A summary of each database search is provided in Supplementary Material 2. Additionally, the ProQuest One Academic database was searched using the same approach to identify any dissertations/theses that may not have been published as scholarly studies. The top 1% of dissertations/theses identified through ProQuest One were screened when sorted by relevance. To identify gray literature, 2 reviewers (M.M. and S.R.) searched Google Scholar using a combination of search terms, with the top 50 results of each search screened. Finally, the references of all included studies were screened using a “forwards and backwards” approach, where both the references of the included studies and studies that have cited the eligible studies (as per Google Scholar) were screened. All search results were exported into Microsoft Excel (Microsoft Corporation, Redmond, Washington, USA) and duplicates manually excluded. Identified studies were screened initially by title and abstract and then by full text. Two independent reviewers (M.M. and S.R.) conducted all searches, with discrepancies resolved by a third reviewer (L.H.).

### Study Selection

Studies were included if they met the following criteria: (1) included individuals with any clinically diagnosed tendinopathy; (2) investigated the participants’ experiences, beliefs, and/or perceptions of either living with or managing their tendinopathy; and (3) utilized at least 1 qualitative methodology for data collection. Tendinopathy was defined as persistent tendon pain and loss of function as a result of mechanical loading by the ICON 2019 Consensus Statement.^17^ Studies that included participants with comorbidities in addition to their diagnosed tendinopathy were included. Where comorbidities were identified, they were documented in the study characteristics table (refer to Suppl. Material 3—study by Ceravolo et al^18^). Letters to the editor, abstracts only, non-peer reviewed articles, and other systematic reviews were excluded.

### Quality Assessment

Two reviewers (M.M. and S.R.) independently assessed the quality of the included studies using the 10-item Joanna Briggs Checklist for Qualitative Research,^19^ with disagreements resolved by a third reviewer (L.H.). Each item was scored as “yes,” “no,” “unclear,” or “not applicable.”^19^ Interrater reliability between the 2 reviewers was analyzed using the kappa statistic (κ) through SPSS V25 software (IBM Corp, Armonk NY, USA). Kappa statistics were interpreted as poor (0.00), slight (0.01-0.20), fair (0.21-0.40), moderate (0.41-0.60), substantial (0.61-0.80), or almost perfect (0.81-1.00).^20^

### Data Extraction and Synthesis

Data were extracted for participant population, demographics, setting, data collection methods, and results, including all themes, participant quotes, and metaphors from each paper. The relevant eMERGe reporting guidelines were incorporated into this systematic review,^12^^,^^13^ accompanied by the 7 phases of meta-ethnography.^21^ The result sections of each included study including quotes, metaphors, themes, and/or concepts were uploaded to NVivo V12 (Lumivero, Massachusetts, USA).^22^ Each document was read repeatedly and juxtaposed against one another using a table grid displaying concepts across all the included studies.^23^ Studies were then translated into one another, and concepts were compared through an interpretive process. A reciprocal translation method was possible given that there were common findings across the included studies.^12^^,^^13^ The translated themes were synthesized to develop a higher order interpretation and aid analysis, interpretation, and communication.^12^^,^^13^^,^^21^

### Confidence in Findings

Confidence in the findings was evaluated using the Grading of Recommendations Assessment, Development, and Evaluation Confidence in the Evidence from Reviews of Qualitative research (GRADE-CERQual).^24^ Two reviewers (M.M. and S.O.) independently completed the evaluation. Four components were used to formulate an overall assessment of confidence in the synthesized qualitative findings: (1) methodological limitations; (2) relevance; (3) adequacy of data; and (4) coherence. Based on these components, an overall rating of confidence was determined as either high, moderate, low, or very low.

### Role of the Funding Source

This research received no specific grant from any funding agency in the public, commercial, or not-for-profit sectors.

## RESULTS

### Search Outcomes

**Figure 1:**
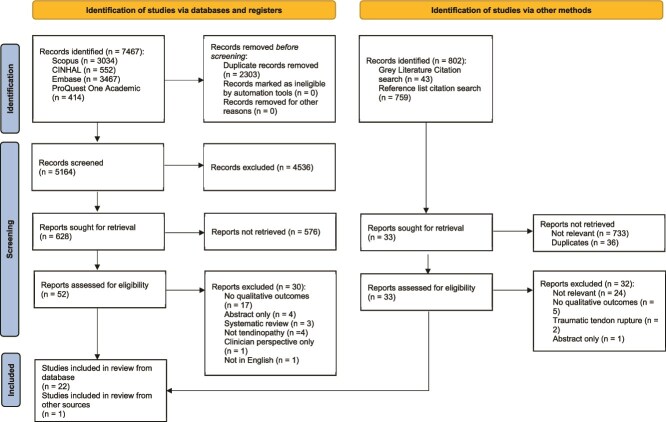
PRISMA 2020 Flow Diagram. From: Page MJ, McKenzie JE, Bossuyt PM, et al. The PRISMA 2020 statement: an updated guideline for reporting systematic reviews. BMJ 2021;372:n71. https://doi.org/10.1136/bmj.n71. For more information, visit: http://www.prisma-statement.org/.

A total of 8269 studies were screened in August 2023 (ie, 7467 from the databases and 802 from the gray literature search). Following removal of 3200 duplicates from the database search, the 2 reviewers screened 4267 studies by title and abstract with 52 identified for full-text review. From the 802 studies identified through the gray literature and citation search, 33 additional studies were retrieved for full-text review. From the 85 full-text studies screened, 62 were excluded, leaving 23 studies included in this review (Figure 1). Supplementary Material 2 provides database search strategies and results.

### Characteristics of Included Studies

Supplementary Material 3 presents the study characteristics. Twelve studies explored rotator cuff tendinopathy,^9^^,^^25–35^ 6 explored Achilles tendinopathy,^8^^,^^18^^,^^36–39^ 2 explored gluteal tendinopathy,^40^^,^^41^ 2 explored lateral elbow tendinopathy,^6^^,^^7^ and 1 study explored people with multiple tendinopathies (eg, proximal hamstrings, insertional Achilles, plantar fasciitis, and patellar).^42^ Semi-structured interviews were undertaken in 16 studies,^7–9^^,^^26^^,^^29^^,^^31^^,^^32^^,^^34–42^ focus group and group settings in 5 studies,^18^^,^^28^^,^^30^^,^^33^^,^^36^ and individual telephone interviews in 2 studies.^25^^,^^30^ The qualitative approaches used in the included studies were relatively consistent. Most studies employed a purely qualitative design, with only 2 studies using a mixed-methods approach.^9^^,^^27^ Interpretive description was a commonly used analysis in 6 of the studies.^7^^,^^8^^,^^34^^,^^36–38^ Thematic analysis was frequently used to identify and interpret patterns in the data. Studies shared similarities in the qualitative research questions related to participants’ understanding and knowledge of tendinopathy,^7^^,^^9^^,^^25–37^^,^^39^^,^^40^^,^^42^ their experience with tendinopathy management,^8^^,^^9^^,^^18^^,^^26–30^^,^^34^^,^^35^^,^^38^^,^^39^^,^^41^^,^^42^ and uncertainty about prognosis and whether their lifestyle will return to normal.^6–9^^,^^18^^,^^28^^,^^29^^,^^33^^,^^34^^,^^37^^,^^38^ The main differences between studies related to the anatomical location (eg, rotator cuff, Achilles, and lateral elbow) or the intervention being explored (eg, exercise programs, pain education, and telehealth). Participant’s ranged from 18^18^ to 86 years old.^26^ The mean age for rotator cuff tendinopathy ranged from 36.5 to 66.0 years,^9^^,^^25–35^ Achilles tendinopathy from 40.0 to 49.2 years,^8^^,^^18^^,^^36–39^ gluteal tendinopathy from 56.0 to 62.4 years,^40^^,^^41^ and lateral elbow tendinopathy from 41.0 to 47.0 years.^6^^,^^7^ The sample sizes ranged from 5^6^ to 92 participants.^18^

### Quality Assessment

Interrater agreement between the 2 reviewers was 95.2% (κ = 0.822, *P* < .001), with 11 discrepancies from 230 decisions. Table 1 presents the quality appraisal for the included studies. Included studies excelled in the first 5 criteria of the 10-item Joanna Briggs Checklist for Qualitative Research, demonstrating strong congruence between philosophical perspective, methodology, research questions, data collection methods, analysis, and interpretation of results. This consistency was observed across all 23 studies, indicating a solid foundation for qualitative research. Most studies (21 out of 23) clearly reported ethical approval from appropriate bodies. Most studies (18 out of 23) adequately represented participants’ voices, ensuring that the research findings accurately reflected the experiences and perspectives of individuals with tendinopathies. Most studies (19 out of 23) provided robust conclusions that reflected the analysis and/or interpretation of the data, demonstrating a strong link between research aims, findings, and conclusions. Only 13 out of 23 studies clearly acknowledged the potential research bias, indicating a need for greater reflexivity in future qualitative research. Most studies (20 out of 23) failed to provide a clear statement locating the researcher culturally or theoretically.

**Table 1:** Quality Appraisal of the Included Studies Using the Joanna Briggs Institute Critical Appraisal Checklist

**Citation**	**1. Congruity Between Philosophical Perspective and Research Methodology?**	**2. Congruity Between Methodology and Research Question/Objectives?**	**3. Congruity Between Methodology and Data Collection Methods?**	**4. Congruity Between Methodology and Representation and Analysis of Data?**	**5. Congruity Between Methodology and Interpretation of Results?**	**6. Statement Locating the Researcher Culturally or Theoretically?**	**7. Is the Influence of the Researcher on the Research Addressed?**	**8. Are Participants Voices Adequately Represented?**	**9. Ethical Approval From an Appropriate Body?**	**10. Do Study Conclusions Represent Analysis or Interpretation of the Data?**
**Acker et al** ^34^	Yes	Yes	Yes	Yes	Yes	Unclear	Yes	Yes	Yes	Yes
**Bateman et al** ^7^	Yes	Yes	Yes	Yes	Yes	Unclear	Yes	Yes	Yes	Yes
**Ceravolo et al** ^18^	Yes	Yes	Yes	Yes	Yes	Unclear	Unclear	Yes	Yes	Unclear
**Cridland et al** ^25^	Yes	Yes	Yes	Yes	Yes	Unclear	Unclear	Unclear	Yes	Yes
**Gillespie et al** ^9^	Yes	Yes	Yes	Yes	Yes	Unclear	Unclear	Yes	Yes	Yes
**Hasani et al** ^36^	Yes	Yes	Yes	Yes	Yes	Unclear	Yes	Yes	Yes	Unclear
**Kiely** ^26^	Yes	Yes	Yes	Yes	Yes	Unclear	Yes	Yes	Yes	Yes
**Lee and Lee** ^6^	Yes	Yes	Yes	Yes	Yes	Yes	Yes	Unclear	Unclear	Yes
**Leung et al** ^42^	Yes	Yes	Yes	Yes	Yes	Unclear	Yes	Yes	Yes	Yes
**Littlewood et al** ^27^	Yes	Yes	Yes	Yes	Yes	Unclear	Yes	Yes	Yes	Unclear
**Malliaras et al** ^29^	Yes	Yes	Yes	Yes	Yes	Unclear	Yes	Yes	Yes	Yes
**Mallows et al** ^37^	Yes	Yes	Yes	Yes	Yes	Unclear	Unclear	Yes	Yes	Yes
**Mc Auliffe et al** ^38^	Yes	Yes	Yes	Yes	Yes	Unclear	Unclear	Unclear	Yes	Yes
**Nyman et al** ^28^	Yes	Yes	Yes	Yes	Yes	Unclear	Unclear	Unclear	Yes	Yes
**Palenius and Nyman** ^30^	Yes	Yes	Yes	Yes	Yes	Unclear	Yes	Yes	Yes	Yes
**Plinsinga et al** ^40^	Yes	Yes	Yes	Yes	Yes	Unclear	Yes	Yes	Yes	Yes
**Powell et al** ^35^	Yes	Yes	Yes	Yes	Yes	Yes	Unclear	Yes	Yes	Yes
**Ryan et al** ^39^	Yes	Yes	Yes	Yes	Yes	Unclear	Yes	Yes	Yes	Yes
**Sandford et al** ^31^	Yes	Yes	Yes	Yes	Yes	Unclear	Yes	Unclear	Yes	Yes
**Sole et al** ^32^	Yes	Yes	Yes	Yes	Yes	Unclear	Yes	Yes	Yes	Yes
**Stephens et al** ^41^	Yes	Yes	Yes	Yes	Yes	Unclear	Yes	Yes	Yes	Yes
**Turner et al** ^8^	Yes	Yes	Yes	Yes	Yes	Unclear	Yes	Yes	Yes	Yes
**Ulack et al** ^33^	Yes	Yes	Yes	Yes	Yes	Yes	Unclear	Yes	Yes	Unclear

### Assessment of Confidence in the Review Findings (GRADE-CERQual)

Key review findings, confidence judgements for each finding, and an explanation of each judgement are presented in Table 2. We had moderate confidence in review findings 1 and 2, mostly due to concerns regarding methodological limitations, adequacy of the data, and relevance, and high confidence in review finding 3 (Table 2). For a more detailed breakdown of the GRADE-CERQual findings see Supplementary Material 4.

**Table 2:** Grading of Recommendations Assessment, Development, and Evaluation Confidence in the Evidence From Reviews of Qualitative Research (GRADE-CERQual) Summary of Findings

**Summary of Review Finding**	**Studies Contributing to the Review Finding**	**Assessment of Confidence in the Evidence**	**Explanation of the Assessment**
1. Patient education and knowledge about tendinopathy: participants with tendinopathy desired to understand why their tendon hurts, seeking clarity in what they were dealing with whether from online resources or health care professionals.	^7^ ^,^ ^9^ ^,^ ^25–37^ ^,^ ^39^ ^,^ ^40^ ^,^ ^42^	Moderate confidence	Moderate concerns regarding methodological limitations and minor concerns regarding coherence, adequacy, and relevance of data.
2. Patient experience with management of tendinopathy: participants with tendinopathy desired to treat their tendon pain. However, they are not sure what’s the best method to do that; whether it’s exercises or passive treatment (eg, massages) or injection therapy or just rest and do nothing.	^8^ ^,^ ^9^ ^,^ ^18^ ^,^ ^26–30^ ^,^ ^34^ ^,^ ^35^ ^,^ ^38^ ^,^ ^39^ ^,^ ^41^ ^,^ ^42^	Moderate confidence	Moderate concerns regarding methodological limitations and minor concerns regarding coherence, adequacy, and relevance of data.
3. Impact of tendinopathy on patient lifestyle: participants with tendinopathy expressed that they were uncertain whether their lifestyle will return to normal with regard to their sports, jobs, or even social activities with their friends and families.	^6–9^ ^,^ ^18^ ^,^ ^28^ ^,^ ^29^ ^,^ ^33^ ^,^ ^34^ ^,^ ^37^ ^,^ ^38^	High confidence	Minor concerns regarding methodological limitations, coherence, adequacy, and relevance of data.

### Synthesis

Three overarching themes were identified that related to a person’s experiences of living with tendinopathy (Table 3).

1) I need to understand why my tendon hurts.
  a) **Descriptors:** patient knowledge and beliefs surrounding causation of tendinopathy
2) I want to fix my tendon, but I don’t know how.
  b) **Descriptors:** patient experience with management, trust in health care professionals, and barriers and enablers to management.
3) I am uncertain whether my lifestyle will return to normal.
  c) **Descriptors:** Impact on lifestyle and future prognosis (positive/negative outlook)

**Table 3:** Representation of the Identified Themes According to Each Included Study Classified by the Anatomical Location of the Tendinopathy

**Citation**	**Anatomical Location of Tendinopathy**	**I Need to Understand Why My Tendon Hurts**		**I Want to Fix My Tendon, but I Don’t Know How**			**I Am Uncertain Whether My Lifestyle Will Return to Normal**	
**Descriptors of the Main Themes**		**Patient Knowledge of Tendinopathy**	**Beliefs Regarding Causation**	**Patient Experience With Management**	**Trust in Health Care Professionals**	**Barriers and Enablers to Management**	**Impact on Lifestyle**	**Future Prognosis (Positive/Negative Outlook)**
Acker et al^34^	**Rotator cuff**	X	X	X			X	X
Cridland et al^25^		X						
Gillespie et al^9^		X	X	X			X	X
Kiely^26^		X		X	X	X		
Littlewood et al^27^		X		X	X	X		
Malliaras et at^29^		X		X	X	X	X	
Nyman et al^28^		X		X		X	X	
Palenius and Nyman^30^		X		X	X			
Powell et al^35^		X		X	X			
Sandford et al^31^		X			X	X		
Sole et al^32^		X						
Ulack et al^33^		X			X	X	X	
Bateman et al^7^	**Lateral elbow**	X					X	
Lee and Lee^6^			X				X	
Ceravolo et al^18^	**Achilles**			X			X	X
Hasani et al^36^		X			X	X		
Mallows et al^37^		X					X	
Mc Auliffe et al^38^			X	X			X	X
Ryan et al^39^		X		X	X	X		
Turner et al^8^			X	X			X	X
Leung et al^42^	**Varied locations** ^*a*^	X		X	X			X
Plinsinga et al^40^	**Gluteal**	X	X					
Stephens et al^41^			X	X				X

^a^ Variety of tendinopathies: proximal hamstring, insertional and mid-portion Achilles, plantar fasciitis, patellar, iliotibial band syndrome, flexor hallucis longus and flexor digitorum longus, insertional adductor longus, insertional infraspinatus tendon, and distal musculotendinous junction of the biceps.

Participant quotes have been included throughout the synthesis from the included studies.

#### I Need to Understand Why My Tendon Hurts

This theme explored patients’ desire to understand tendinopathy and its etiology, which emerged in 18 of the 23 studies.^7^^,^^9^^,^^25–37^^,^^39^^,^^40^^,^^42^ Participants accessed information from various sources including online resources and health care professionals,^7^^,^^9^^,^^25^^,^^29^^,^^34^^,^^39^^,^^40^ with information from health care professionals seen to be “superior” and more “trustworthy” compared to online sources in some studies.^7^^,^^9^^,^^25^^,^^29^^,^^34^

*“I did Google once I was diagnosed as having Tennis Elbow. You’re kind of curious to know what exactly it is.” (Participant DER011)* ^7^

*“It was the clarity and the confidence in what and how he was dealing with it, which I found the most useful.” (Participant 5)* ^37^

Patient education provided “clarity” and “confidence” that their treating clinician understood their condition, which enabled “trust” and allowed patients to ask questions to improve their understanding of their condition.^29^^,^^33^^,^^37^

*“I felt reassured that it wasn’t an injury as such. And by moving I wasn’t going to make it worse.” (Quote 21)* ^34^

Patient education that included a definitive clinical diagnosis contributed to “feelings of relief” that the patients’ pain may be explained by pathological findings.^40^ In addition, patient education reduced the “fear” and common misconceptions associated with performing activities during recovery.^8^^,^^9^^,^^26^^,^^28^^,^^32^^,^^33^

Participants’ desire to understand the cause of their tendon pain emerged in 7 of the 23 studies.^6^^,^^8^^,^^9^^,^^34^^,^^38^^,^^40^^,^^41^

*“To this day [I] don’t really know what the actual [cause of the pain is], other than the fact that I know (…) where the instances occurred.” (Quote 6)* ^9^

Some patients attributed their tendinopathy to internal/covert factors including “weakness,” “lack of fitness,” “running style,” or “footwear,”^8^^,^^38^ while others believed external/overt factors such as “over-training,” “overuse,” and “lack of recovery time” were the cause.^8^^,^^38^

*“So, I feel that I probably over trained. Not so much leading up to the run, it was more I didn’t recover and allow myself time to recover afterwards and I just pushed it a little bit too far.” (Participant 10)* ^8^

Participants within studies that included medical imaging linked the cause of their tendinopathy to specific pathological findings.^8^^,^^25^^,^^29^^,^^33^^,^^40^^,^^41^ However, some participants expressed feelings of “confusion” due to limited “information or explanation of the cause” of their tendinopathy based on the imaging results.^8^^,^^9^^,^^32–34^ In comparison, when participants had a clear and definitive diagnosis based on medical imaging, they expressed “relief” that they knew the cause of their tendon pain.^25^^,^^28^^,^^29^^,^^33^^,^^40^^,^^41^ Most participants who received imaging believed it was important and provided an “accurate” diagnosis.^29^^,^^33^^,^^40^

*“I was quite glad that I got the… MRI of the hip… I felt that was something that I gained from doing it…because I was then able to show it to my local doctor…and I suppose that helps to rule out certain conditions” (Participant 14)* ^40^

#### I Want to Fix My Tendon, But I Don’t Know How

This theme related to the process of seeking, receiving, and implementing treatment for tendinopathy. This included factors during this process that both improved and hindered treatment, such as the quality of the patient-clinician relationship**.**

Participants’ experience with management of tendinopathy appeared in 14 of the 23 studies.^8^^,^^9^^,^^18^^,^^26–30^^,^^34^^,^^35^^,^^38^^,^^39^^,^^41^^,^^42^ Three studies reported that patients with tendinopathy were prescribed strength training exercises either as part of a self-management program or as part of individual or group therapist-led setting.^8^^,^^27^^,^^37^

*“The strengthening exercises got rid of my pain. It was really bizarre process to…starting to lift weights slowly and doing certain things, like increasing my range in my shoulder with the strength exercises, the pain would just go.” (Participant: Lisa)* ^35^

Patients reported disengagement from rehabilitation when provided with generic information or treatment.^25^^,^^27^ Similarly, some participants with rotator cuff tendinopathy expressed frustration when management was perceived as simplistic and ineffective.^27^

*“Unexpectedly, disquiet was expressed about the simplicity of the intervention and hence its lack of potential effectiveness… to cap it all it’s such a simple exercise… I just came out thinking waste of time.” (Participant 29)* ^27^

Two studies reported that patients expected “passive treatment” (eg, massage therapy and dry needling) as part of physical therapist management for their tendinopathy with the belief their symptoms could be managed without much effort or pain associated with exercise.^8^^,^^30^

*“Obviously, massage, anything to loosen up my calf, really. So, massage work or acupuncture on my calves.” (Participant 9)* ^8^

Four studies discussed participants’ experiences of injection therapy as part of tendinopathy management.^7^^,^^28^^,^^29^^,^^41^ In 1 study, most participants had received previous corticosteroid injections as a first line treatment and of these many reported short-term relief.^41^

*“I had a couple of injections; you know the injections they do for the steroids and stuff. That helped me. It went away for a couple of years. I didn’t have any pain but after it came back again.” (Participant: BHX004)* ^7^

Patients viewed steroid injections as a temporary treatment, where long-term efficacy is uncertain.^29^ In contrast, some patients believed cortisone injections were going to be effective long-term, as they are given by a physician.^28^

*“… I’ve subsequently learnt that you never have cortisone ever, because it’s just basically is a Band-Aid, and it can mask the pain and you can do things that cause further damage.” (Participant 2)* ^29^

Patient trust in health care professionals was highlighted in 10 of the 23 included studies.^26^^,^^27^^,^^29–31^^,^^33^^,^^35^^,^^36^^,^^39^^,^^42^ Support provided by the health care professional that encouraged self-management through exercise was framed in positive way, even when progress was slow.^25^^,^^35^^,^^39^

*“I remember doing the six-week check-in with you, where the function had improved a lot and definitely that would have just given me great confidence that this was working and to keep on going.” (Participant 11)* ^39^

Having regular contact with a health care professional to obtain feedback, even if via telehealth, allowed participants to adhere to their exercises.^36^ Patients believed that supportive and responsive health care professionals, including those available outside of appointment times (eg, phone calls), facilitated progress with exercises and provided confidence in receiving optimal management.^27^^,^^39^ In addition, some participants valued the health care professionals’ qualifications and reputation building trust in the management process.^29^^,^^39^

Lack of motivation or self-discipline to management (including exercise) appeared in 8 of the 23 studies.^26–29^^,^^31^^,^^33^^,^  ^36^^,^^39^

*“The big problem was lack of motivation.” (Participant 62)* ^31^

Some participants reported that a lack of motivation was still present, despite having the time to do the exercise.^31^ Many participants acknowledged pain during exercise to be a barrier to rehabilitation,^26^^,^^31^^,^^33^ with some stating they need to push themselves to do the painful exercise.^26^ In contrast, some participants perceived that pain equated to damage^33^ and others ceased exercise because they were afraid that exercise would make symptoms worse.^31^^,^^38^

*“There was one exercise that I was given earlier that I thought at the time might have made things worse, so I stopped doing it” (Participant 69)* ^31^

Cost and time of seeking medical treatment was also perceived to be a barrier by many participants, especially those who were self-employed.^9^^,^^28^^,^^36^

*“Literally just time allocation in what I do. Because I do not work for somebody, so I do not have a nine to five job.” (Participant 3)* ^36^

Participants expressed desire for the intervention to fit within their work schedule and to recover quickly and minimize absence from work.^28^^,^^36^ Participants found it difficult to balance their work and rehabilitation commitments, especially when completing low-intensity high-repetition exercise which required time to complete.^36^ Some patients reported that their rehabilitation took longer than expected.^28^ The simplicity and time to complete exercise were enablers for some participants to engage in rehabilitation.

*“The elastic band, I think, is worth the money and I liked being able to go up the levels…I felt like I was progressing.” (Participant 60)* ^31^

Participants believed that exercises should be personalized, simple, convenient, and easy to perform, with many participants preferring a simple exercise (eg, double leg calf raise) with a clear explanation compared exercises that were complex to perform.^36^^,^^42^

*“It’s easier to do the exercises when you can feel a definite benefit...” (Participant 66)* ^31^

In contrast, other participants thought simple exercises may be ineffective.^27^

#### I Am Uncertain Whether My Lifestyle Will Return to Normal

This theme explored patients’ uncertainty regarding their prognosis with tendinopathy and their ability to resume normal daily activities. Patient perceptions varied, with some believing the condition was not serious, while others were concerned about recovery due to persisting symptoms.

The impact of tendinopathy on participants’ lifestyle appeared in 11 of the 23 studies.^6–9^^,^^18^^,^^28^^,^^29^^,^^33^^,^^34^^,^^37^^,^^38^ Some participants described their pain as severe^6^ or “fierce,”^28^ while others stated that pain was “unpredictable,” “annoying,” and “fluctuating.”^9^ Many participants stated their pain severity affected their activities of daily living and physical mobility, which negatively impacted their quality of life.^6–8^^,^^29^^,^^34^^,^^37^^,^^38^ Participants reported feeling depressed due to the negative impact that their symptoms had on their self-esteem and their interactions within their social networks.^6^^,^^18^^,^^37^

*“My whole social network is full of runners, so, rather than get cranky, I’ve stopped going, so, you’re actually withdrawing from the social networks.” (Participant ID not provided)* ^18^

Participants described the negative impact of tendinopathy on their “self-identity” and felt frustrated that they cannot be the person that they want to be^37^ or do the things they want to do.^6^^,^^18^ Participants with Achilles tendinopathy noted they spent less time socializing and seeing their friends,^18^ with some reporting their social activities (eg, going for walks or playing with their children) and their sports (eg, running) were greatly impacted or completely stopped.^8^ In addition, participants expressed “fear” that they could not support themselves and their families financially, especially if they worked in manual labor jobs.^33^ Many participants felt the need to take medications to combat the pain, despite the fear of side effects.^28^^,^^29^

*“From a career perspective it was having a constant impact, constant pain, difficult to perform the necessary tasks and then on a day-to-day perspective I was having difficulty with things like carrying my shopping home.” (Participant: DER006)* ^7^

Future prognosis appeared in 7 of the 23 studies, with some participants reporting a positive outlook while others reported a negative outlook.^8^^,^^9^^,^^18^^,^^34^^,^^38^^,^^41^^,^^42^

*“These issues I’d say they are quite resistant so far, for quite a long time, so if they completely disappear, that would be great, but I’m not sure if this is going to happen.” (Participant 10)* ^42^

Participants with Achilles tendinopathy were optimistic about their recovery and returning to their previous level of function,^8^^,^^38^ suggesting that prescribed exercise was an important driver for future improvement.^8^^,^^34^ However, other participants reported a perceived poor prognosis, due to their perceptions of pathology, chronicity, and genetics of tendinopathy.^8^ Participants with gluteal tendinopathy also had mixed views on their recovery with some confident they would recover in about 6 months’ time with beliefs their condition was not serious, while others expressed uncertainty of recovery due to persisting symptoms.^41^ In addition, some participants with rotator cuff tendinopathy expressed concerns their activities might cause permanent damage, with beliefs that pain will not subside.^9^

*“I thought that I would be living with um, the pain (…) and that it wasn’t really controllable and now the flip side of that is now I think it probably is controllable and um, that my shoulder injury is really pretty minor and with a little bit of exercise and quite probably the right attitude it is completely manageable.” (Quote 22)* ^34^

Participants with a variety of tendinopathies who did not complete their course of treatment (ie, radial extracorporeal shockwave therapy) had uncertainty that their tendinopathy condition will be cured.^42^

## DISCUSSION

### Summary

This review synthesized 23 qualitative studies that investigated the lived experiences of patients with tendinopathy, highlighting 3 key themes: (1) I need to understand why my tendon hurts; (2) I want to fix my tendon, but I don’t know how; and (3) I am uncertain whether my lifestyle will return to normal. Generalizing these themes to all patients with tendinopathy warrants caution, given that 11 of the 23 studies had rotator cuff pathology and not all tendons have been studied. That said, our identified themes show broad consistency with reviews exploring the lived experience of people with non-specific low back pain (NSLBP), fibromyalgia, and knee osteoarthritis.^43–45^ For example, individuals with NSLBP, fibromyalgia, and knee osteoarthritis also sought a clear explanation of their pain and a causal understanding of their condition,^43–45^ which for patients with NSLBP was a pre-requisite for engaging with treatment.^46^ While our findings, and that of others^43–45^ suggest individuals with tendinopathy, NSLBP, fibromyalgia, and knee osteoarthritis seek explanations for their symptoms and want to make sense of their condition, we cannot conclusively state that this applies equally to all patients with musculoskeletal conditions. Our findings indicate potential benefits for patients with tendinopathy where a clinician provides an evidence-based explanation of the diagnosis and pathology of the tendon, supports the patient through appropriate rehabilitation (ie, progressive strengthening program), and identifies and dispels fears or psychosocial impacts associated with tendinopathy. However, it is essential to recognize that the effectiveness of this approach may vary depending on the specific type of tendinopathy and individual patient characteristics.

### Theme 1: I Need to Understand Why My Tendon Hurts

Patients’ desire to understand their musculoskeletal condition is well documented in qualitative research^47^ and was the most common theme from our findings, identified in 78% of included studies. Consistent with a patient-centered care framework,^48^ improving patients’ understanding of their tendinopathy, within a biopsychosocial context, may help patients make sense of their condition, alleviate fears, and empower individuals to actively participate in their recovery. Similarly for individuals with NSLBP, obtaining a diagnosis provided relief, especially if they fear serious underlying causes.^46^ However, it may be important to emphasize to patients that medical imaging is typically not required in most cases of tendinopathy,^17^ as pain does not always correlate with structural pathology.^49^ Educating patients about the complex interplay of factors contributing to tendon pain, such as overuse and tendon biomechanics, may help individuals adopt a more holistic perspective of the condition, irrespective of the structural state of their tendon.

Early and focused patient education can enhance the patient-clinician relationship by improving trust.^50^ This improved trust may enhance patient’s adherence to treatment plans, make lifestyle modifications, and engaging in appropriate exercises without fear.^51^ As observed in our included studies,^8^^,^^38^ a misunderstanding or lack of education may lead to uncertainty and strain patient-clinician relationships, especially when communication is poor, and patient-clinician beliefs do not align.^52^ Effective patient education may lead to informed decision-making, better adherence to treatment, and improved rehabilitation outcomes. Effective patient education may lead to informed decision-making, better adherence to treatment, and improved rehabilitation outcomes.

### Theme 2: I Want to Fix My Tendon, But I Don’t Know How

Patients’ experiences with treatment varied widely, with some patients preferring passive treatment (eg, manual therapy and ultrasound) over progressive strengthen exercises.^8^^,^^30^ While short-term improvements in pain have been observed using manual therapy and massage,^53^^,^^54^ level 1 evidence shows progressive strengthening exercises are more effective for both upper and lower limb tendinopathy.^55–57^ Therefore, patients should be educated regarding the long-term benefit of progressive strengthening exercise and reassured that for most people symptoms will subside over time.

Our findings emphasize the importance 2-way communication to understand patients’ experiences and expectations and to co-design rehabilitation programs aligned with patient goals for optimal outcomes. By prioritizing patient understanding and providing thorough explanations, health care professionals empower patients to actively participate in their care decisions, aligning with a patient-centered care framework.^58^^,^^59^

Our findings highlighted several barriers and facilitators to engaging in rehabilitation. For example, patient expectations, lack of time, poor self-discipline, and fear of further damage likely impact on patients’ motivation and willingness to engage in rehabilitation. Patient expectations significantly influence clinical outcomes in musculoskeletal conditions.^60^ Positive expectations improve outcomes and motivate rehabilitation, while negative expectations lead to poorer outcomes.^60^ Patient expectations can have a major influence on clinical outcomes in musculoskeletal conditions. Positive expectations are associated with improved outcomes and motivate engagement in rehabilitation, while negative expectations are associated with poorer outcomes.^61^ Therefore, we advocate for clinicians to foster positive expectations and reassure patients where negative expectations are identified. Consistent with our findings, a lack of time, poor self-discipline, and fear of further damage are considered barriers to engagement in exercise rehabilitation in musculoskeletal conditions such as knee osteoarthritis.^62^ Whilst engaging patients in exercise programs can be difficult, our findings suggest that personalized, yet simple exercises are preferred by patients. In addition, improving patient knowledge, increasing social support, and goal setting can further help adherence to exercise programs.^62^

### Theme 3: I Am Uncertain Whether My Lifestyle Will Return to Normal

Our findings revealed that tendinopathy negatively impacted patients’ lifestyle. Studies within our review reported patients had limitations and/or restrictions in doing things they enjoy, which negatively affected their quality of life.^8^^,^^18^^,^^33^ Consistent with our findings, other qualitative studies on knee osteoarthritis^63^ and chronic lower back pain,^64^ found patients were uncertain about returning to normal activities due to pain and fear of causing “damage”. Within patient-centered care, addressing patients’ uncertainty about returning to normal life is crucial, as it acknowledges the potential psychosocial impact on their recovery.^65^^,^^66^ Effective patient education has potential clarify uncertainties and negative beliefs about returning to normal life, improving patient outcomes and promoting positive health behaviors.^67^

### Clinical Implications

Patient experiences of persistent tendon pain may not be dissimilar to other forms of persistent musculoskeletal pain.^68^ However, this meta-ethnography offers additional insights into the context of tendinopathy for health care professionals. In line with the 3 key themes identified in this study, health care professionals should aim to: (1) provide individualized, evidence-based education to clarify the relationships between tendon pathology, pain, and function; (2) promote tendon loading through physical activity and exercise to dispel fears, build rapport, increase adherence to progressive strengthening, and reduce reliance on passive therapies; and (3) recognize and incorporate the psychological and/or social impacts of tendon pain for a holistic, patient-centered approach.

#### Recommendation 1. Improve Patient Understanding

Improving patient knowledge is an important aspect of effective health care delivery^69^^,^^7^^,0^, and lacking patient understanding of their health condition may lead to poor health outcomes and reduced treatment adherence.^71^^,^^72^ To address this, several evidence-based approaches have been identified to improve patients’ understanding of tendinopathy. First, health care professionals should provide pain and pathoanatomic patient education, encourage evidence-based exercise therapy to improve tendon load capacity, and use additional therapies like manual therapy and massage as adjuncts.^73^ Second, patient-centered communication improves understanding by engaging patients in discussions and addressing their specific concerns.^70^ Third, using clear language and visual aids (ie, models, pictures, videos, brochures, and online resources) can enhance patient understanding of their tendinopathy condition.^69^^,^^72^ Additionally, online patient education platforms^74^ serve as valuable tools for health education and self-management. Finally, the teach-back method, where patients explain information about their condition back to health care professionals, significantly improves comprehension and retention of medical information.^75^

#### Recommendation 2. Dispel Fears

To minimize poor recovery, negative beliefs and psycho-social factors should be addressed. First, health care professionals may use open-ended questions to encourage patients to express concerns regarding tendinopathy. Second, employing validated questionnaires (eg, Tampa Scale for Kinesiophobia^76^ and Pain Catastrophizing Scale^77^) can provide a structured approach to identify areas of concern and allow an avenue to seek assistance from mental health professionals, when necessary. Lastly, visual aids, analogies, and clear language may be used to dispel fears, while using the teach-back method to confirm patient comprehension.^78^

#### Recommendation 3. Provide Reassurance

Health care professionals should provide reassurance that gradual exercise is important to improve tendon strength and reduce pain.^79^ There are several recommendations for clinicians to provide patients with reassurance regarding their tendinopathy management. First, active listening is important for reassuring anxious patients^80^ and can be demonstrated by maintaining eye contact, nodding, and providing verbal acknowledgments.^80^ Second, shared decision-making has been shown to reassure patients and reduce anxiety in tendinopathy.^68^ Health care professionals should discuss tendinopathy treatment options and explain the pros and cons, involving the patient in choosing the preferred approach for their lifestyle.^72^ Third, patients feel reassured by appropriate non-verbal behaviors such as maintaining eye contact, positive facial expressions, and confident body posture.^81^ By implementing these strategies, health care professionals may enhance patient engagement and improve overall health outcomes.

### Methodological Considerations

There are several methodological considerations when interpreting the results of this review. Despite a systematic search strategy, it is possible that relevant studies were missed. Not all studies thoroughly explored the lived experience of patients with tendinopathy, with some studies electing to focus on experiences with a specific treatment,^40^^,^^42^ which might limit generalizability to other treatment modalities. In addition, nearly half of the studies reported on rotator cuff tendinopathy (11/23), and not all tendinopathies had qualitative studies to include (eg, patellar and medial elbow), limiting generalizability of our findings across all tendons. We assessed the confidence in our review findings using the GRADE-CERQual framework^24^ and considered it likely that our review findings are a good representation of the experiences of individuals living with tendinopathy. However, varying methodological rigor in qualitative studies can affect the validity and reliability of meta-synthesis. Additionally, synthesizing qualitative data across multiple studies may oversimplify or decontextualize nuanced findings, compromising the rich, contextual depth that characterizes qualitative research. The main author (M.M.) is a physical therapist in Australia, and the impact of his clinical experiences likely contributed to the interpretation of the results. Our multidisciplinary team of physical therapists and psychologists enabled a nuanced understanding of the psychological and physiological factors in tendinopathy management. Including studies in other languages may have provided additional insights. Our multidisciplinary team of physical therapists (M.M., S.R., L.H., and S.O.), and psychologists (C.D. and A.R.) enabled a comprehensive and nuanced understanding of the psychological and physiological factors in tendinopathy management. Finally, inclusion of studies in languages other than English may have provided additional insights.

## CONCLUSIONS

This review provides valuable insight into the impact of tendinopathy on patients’ lives and their experiences with rehabilitation. Qualitative synthesis from 23 studies identified 3 themes: (1) I need to understand why my tendon hurts; (2) I want to fix my tendon, but I don’t know how; and (3) I am uncertain whether my lifestyle will return to normal. Patients sought clarity regarding the cause of tendinopathy and expressed varied beliefs regarding optimal management, where pain and pathoanatomical education should be targeted at their current level of knowledge. Tendinopathy may affect psychological (eg, fear) and social (eg, limited participation) wellbeing, requiring clinician discussion to minimize negative outcomes. Future studies should explore effective patients’ education strategies to improve adherence to evidence-based interventions and achieve optimal patient outcomes.

## Supplementary Material

2024-0132_R2_Supplementary_Material_pzaf060

## Data Availability

The data are available from the corresponding author upon request.

## SYSTEMATIC REVIEW REGISTRATION

The protocol was prospectively registered with the International Prospective Register of Systematic Reviews (PROSPERO; CRD42022313718).
