# Supplementary material for: Exploring the Beliefs, Perceptions, and Experiences of Individuals With Tendinopathy: A Systematic Review and Meta-Ethnography of Qualitative Studies
Source: Phys Ther. 2025 Apr 28;105(7):pzaf060. doi: 10.1093/ptj/pzaf060 (PMC12212419; doi:10.1093/ptj/pzaf060)
Supplement: 2024-0132_R2_Supplementary_Material_pzaf060 [file 2024-0132_r2_supplementary_material_pzaf060.pdf]

**Supplementary Material 1.** The eMERGe meta-ethnography reporting guidance table[12]

| Phase | Criteria Number | Criteria Heading | Reporting Criteria |
|---|---|---|---|
| 1. Selecting meta-ethnography and getting started | 1 | Rationale and context for meta-ethnography | Describe gap in research or knowledge to be filled by the meta-ethnography, and the wider context of the meta-ethnography |
| | 2 | Aim(s) of the meta-ethnography | Describe meta-ethnography aim(s) |
| | 3 | Focus of the meta-ethnography | Describe the meta-ethnography review question(s) or objectives |
| | 4 | Rationale for using meta-ethnography | Explain why meta-ethnography was considered the most appropriate qualitative synthesis methodology |
| 2. Deciding what is relevant | 5 | Search strategy | Describe rationale for literature search strategy |

| Phase | Criteria Number | Criteria Heading | Reporting Criteria |
|---|---|---|---|
| | 6 | Search processes | Describe how literature searching was conducted and by whom |
| | 7 | Selecting primary studies | Describe study screening, selection process and who was involved |
| | 8 | Outcome of study selection | Describe results of searches and screening |
| 3. Reading included studies | 9 | Reading and data extraction approach | Describe reading and data extraction methods |
| | 10 | Presenting characteristics of included studies | Describe characteristics of included studies |

| Phase | Criteria Number | Criteria Heading | Reporting Criteria |
|---|---|---|---|
| 4. Determining how studies are related | 11 | Process for determining how studies are related | Describe the methods and processes for determining how the included studies are related (Which aspects of studies were compared AND How the studies were compared) |
| | 12 | Outcome of relating studies | Describe how studies relate to each other |
| 5. Translating studies into one another | 13 | Process of translating studies | Describe methods of translation:<br>• Describe steps taken to preserve the context and meaning of the relationships between concepts within and across studies<br>• Describe how the reciprocal and refutational translations were conducted<br>• Describe how potential alternative interpretations or explanations were considered in the translations |
| | 14 | Outcome of translation | Describe interpretive findings of translation |

| **Phase** | **Criteria Number** | **Criteria Heading** | **Reporting Criteria** |
|---|---|---|---|
| 6. Synthesizing translations | 15 | Synthesis process | Describe methods to develop overarching themes (synthesised translations) |
| | 16 | Outcome of synthesis process | Describe the new theory, conceptual framework, model, configuration, or interpretation of data developed from the synthesis |
| 7. Expressing the synthesis | 17 | Summary of findings | Summarize main interpretive findings |
| | 18 | Strengths, limitations, and reflexivity | Reflect on strengths and limitations of synthesis |
| | 19 | Recommendations and conclusions | Describe implications of the synthesis |

**Supplementary Material 2.** Database search strategies and results (August 2023)

| Database | Date accessed | Search strategy | Additional Limits | Results |
|---|---|---|---|---|
| Scopus | 03/08/2023 | ( ( TITLE-ABS-KEY ( experience ) OR TITLE-ABS-KEY ( belief ) OR TITLE-ABS-KEY ( perception ) ) ) AND ( ( TITLE-ABS-KEY ( tendinosis ) OR TITLE-ABS-KEY ( tendinitis ) OR TITLE-ABS-KEY ( tendinopathy ) OR TITLE-ABS-KEY ( tendonopathy ) OR TITLE-ABS-KEY ( epicondylalgia ) OR TITLE-ABS-KEY ( epicondylitis ) OR TITLE-ABS-KEY ( jumpers AND knee ) OR TITLE-ABS-KEY ( tennis AND elbow ) OR TITLE-ABS-KEY ( golfers AND elbow ) OR TITLE-ABS-KEY ( gluteus ) OR TITLE-ABS-KEY ( patella ) OR TITLE-ABS-KEY ( rotator AND cuff ) ) ) | No additional limits on manuscript | 3034 |
| CINAHL | 03/08/2023 | (tendinosis OR tendinitis OR tendinopathy OR tendonopathy OR epicondylalgia OR epicondylitis OR jumpers knee OR tennis elbow OR golfers elbow OR gluteus OR patella OR rotator cuff) AND (S1 AND S2) | No additional limits on manuscript | 552 |
| EMBASE | 03/08/2023 | #1 experience OR belief OR perception<br>#2<br>tendinosis OR tendinitis OR tendinopathy OR tendonopathy OR epicondylalgia OR epicondylitis OR 'jumpers knee' OR (tennis AND elbow) OR (golfers AND elbow) OR gluteus OR patella OR (rotator AND cuff)<br>#1 AND #2 | No additional limits on manuscript | 3467 |

| | | | | |
|---|---|---|---|---|
| ProQuest One Academic | 03/08/2023 | (experience OR belief OR perception ) AND ((tendinosis OR tendinitis OR tendinopathy OR tendonopathy OR epicondylalgia OR epicondylitis OR (jumpers knee) OR (tennis elbow) OR (golfers elbow) OR gluteus) OR (patella OR (rotator cuff))) | Top 1% of dissertations and theses were screened when sorted by relevance | 414 |

**Supplementary Material 3.** Characteristics of eligible studies reviewed

| **Citation and Country** | **Research Aims** | **Anatomical region and Sample Population** | **Methods** | **Summary of findings (Themes identified by authors in each study)** |
|---|---|---|---|---|
| Acker et al.[34]<br><br>New Zealand | To explore the perspectives and experiences of participants with RCRSP who had completed a programme of Pain Neuroscience Education informed pragmatic physiotherapy. | Condition: Rotator cuff related shoulder pain<br><br>Participants: N =10 [5 males, 5 females], mean (SD) age = 62 (NR) years; duration of symptoms – median (range) = 12 (5-300) months, SPADI median (range) = 43 (11.5-73.1), PSEQ median (range) = 48 (32-60) | Qualitative, interpretive description design was performed using semi-structured interviews | Four themes identified:<br><br>1. Patient Beliefs<br>2. Rapport and Relationship<br>3. Perspective and Understanding of the Resources<br>4. Empowerment: My Shoulder into the Future |
| Bateman et al.[7]<br><br>United Kingdom | To explore the lived experience of people with lateral elbow tendinopathy and its impact on everyday life. | Condition: Lateral elbow tendinopathy<br><br>Participants: N = 17 [9 males, 8 Females], mean (SD) age = 47 (NR); duration of symptoms – median (range) 6 (2-36) months, baseline PRTEE median (range) 47 (18.5 – 93) | Qualitative, interpretive description design was performed using semi-structured interviews | Four themes identified:<br><br>1. Cause of onset<br>2. Impact on everyday life<br>3. Self-help and understanding of the condition<br>4. Healthcare experiences |
| Ceravolo et al.[18]<br><br>Australia | To explore the quality-of-life and experiences of people with Achilles tendinopathy | Condition: Achilles Tendinopathy<br><br>Participants: N = 92 [49 males, 43 females], mean (SD) age – NR (NR); duration of symptoms – median (range) 13.9 (NR) months; VISA-A Score (/100) median (range) = 72.3 (NR)<br><br>Out of the 92 individuals who did the online survey, 11 individuals participated in focus groups. **5 participants had** | Mixed-methods research used the 8-dimension Assessment of Quality-of-Life (AQoL-8D), focus groups and grounded theory analysis. | Seven themes identified:<br><br>1. Adapting lifestyle<br>2. Living with the condition<br>3. Changes in mental well-being<br>4. Conflict with identity<br>5. Frustration<br>6. Individuals' experiences<br>7. Changes in social well-being |

| | | | | |
|---|---|---|---|---|
| | | **comorbidities; Hashimoto disease (n = 1), low back pain (n = 1), depression (n = 1), and high blood pressure (n = 2).** | | |
| Cridland et al.[25]<br>Australia | To explore the experiences and perspectives of people with rotator cuff related shoulder pain about education for their condition. | Condition: Rotator cuff related shoulder pain<br>Participants: N =8 [3 males, 5 females]; mean (SD) age = NR; duration of symptoms – median (range) = 30 (NR) months. | Inductive qualitative design using individual telephone interviews | Three themes identified:<br>1. A therapeutic alliance leads to trust of education<br>2. Educational information should be individualised and practical<br>3. Delivery of educational interventions should be varied and multi-modal |
| Gillespie et al.[9]<br>New Zealand | To explore beliefs about the cause of pain in individuals with persistent rotator cuff-related pain, as well as the experiences of the effect of pain on their daily lives. | Condition: Rotator cuff related pain<br>Participants: N =10 [5 males and 5 females]; median (SD) age = 58 (NR) years; duration of symptoms – median (range) = 11 (4-240) months | Mixed methods design, using semi-structured interviews and validated outcome questionnaires. | Four themes identified:<br>1. Understanding the pain<br>2. It affects everything<br>3. Pain-associated behaviours<br>4. Emotional responses and the future |
| Hasani et al.[36]<br>Australia | To explore the experience of participants and physiotherapists with gym-based exercise interventions for Achilles tendinopathy monitored via videoconference. | Condition: Achilles tendinopathy<br>Participants: N =8 [sex NR]; mean (SD) age = 46.1 (NR) years; duration of symptoms – median (range) = 47.8 (3-240) months; VISA-A (/100) median (range) = 49.8 (17-74) | Qualitative, interpretive description design was performed using semi-structured interviews | Five themes identified:<br>1. Acceptability of telehealth<br>2. Enablers to adherence with telehealth<br>3. Barriers to adherence with telehealth<br>4. Enablers to adherence with the exercise intervention<br>5. Barriers to adherence with the exercise intervention |
| Kiely et al.[26]<br>Ireland | Qualitative study part of the thesis - To explore | Condition: Rotator cuff tendinopathy | Qualitative, inductive thematic analysis design | Three themes identified:<br>1. What patients value from treatment |

| | | | | |
|---|---|---|---|---|
| | participants experiences of and preferences for both formats of group based and individual physiotherapy treatments | For the qualitative study: Participants: N =10 [2 males, 8 females]; mean (SD) age (SD) – 66 (NR); duration of shoulder – median (range) 10.0 (NR) for Group exercise, 20.5 (NR) for Individual treatment | using semi-structured interviews | 2. Engagement with exercise during & after treatment<br>3. Characteristics of successful outcome |
| Lee and Lee.[6]<br><br>Korea | To investigate tennis elbow patients' experience with alternative therapy use and reveal their alternative therapy experience. | Condition: Tennis elbow<br><br>Participants: N =5 [3 males, 2 females], mean (SD) age = 41 (NR) years; duration of symptoms – median (range) = 16 (3-36) months | Qualitative, inductive, descriptive design using in depth interviews | Eight themes identified:<br><br>1. Sense of loss<br>2. Sense of emptiness/desperation<br>3. Trend (Time of exposure/speed of pain)<br>4. Desire<br>5. Supportive system<br>6. Active response<br>7. Contingency<br>8. Self-conformity / self-adaptation |
| Leung et al.[42]<br><br>United Kingdom | To understand patients' overall perspective of radial extracorporeal shockwave therapy (rESWT) to manage their tendinopathy. | Condition: Variety of included tendinopathies (i.e., proximal hamstring, insertional and mid-portion Achilles, plantar fasciitis, patellar, ITB syndrome, FHL and FDL, insertional adductor longus, insertional infraspinatus td and distal MTJ biceps)<br><br>Participants: N =11 [8 males, 3 females]; mean (SD) age = 40.5 years (NR); duration of symptoms – median (range) = NR (2-240) months | Qualitative, thematic design using 'Framework Analysis' with semi-structured face-to-face interviews | Four themes identified:<br><br>1. Choice of rESWT<br>2. Preconceptions of rESWT<br>3. Experience of rESWT<br>4. Current views of rESWT |
| Littlewood et al.[82]<br><br>United Kingdom | To explore potential barriers with participants involved in a pilot randomised controlled trial | Condition: Rotator cuff tendinopathy<br><br>Participants: N =6 [3 males, 3 females]; mean (SD) age = 64.7 (NR) years; duration of | Qualitative design within the framework of a mixed methods research design using individual | Three themes identified:<br><br>1. Expectations and preferences<br>2. Characteristics of an unsuccessful outcome |

| | | | | |
|---|---|---|---|---|
| | (RCT) investigating a self-managed loaded exercise intervention. | symptoms – median (range) = 38.6 months (range 3 to 168); SPADI median (range) = 19.7 (3.1-42.3) | semi-structured interviews | 3. Characteristics of a successful outcome |
| Malliaras et al.[29]<br><br>Australia | To explore the decision-making processors of people who have undertaken surgery for rotator cuff related shoulder pain (RCRSP). | Condition: Rotator cuff related shoulder pain<br><br>Participants: N =15 [8 males, 6 females, 1 prefer not to identify gender]; mean (SD) age = 54.9 (NR) years; duration of symptoms – mean (range) = NR (15-52) weeks | Qualitative, thematic analysis using in depth semi-structured interviews | Six themes identified:<br><br>1. Needing to get it done: “It was necessary to remedy the dire situation”<br>2. Non-surgical treatment experience: “I knew that I’d done all I could”<br>3. Mechanical problem: “Physio’s not going to repair a torn tendon”<br>4. Trust in medical professionals: “If they told me that I needed to swallow a thousand spiders, I would have done it”<br>5. Varied information sources: “Dr Google played a big part in it”<br>6. Organisational barriers: “It was absolutely useless, my insurance” |
| Mallows et al.[37]<br><br>United Kingdom (mainly) and Australia | To gain an insight into patients’ experiences of participating in an exercise-based rehabilitation programme for Achilles Tendinopathy (AT) | Condition: Achilles tendinopathy<br><br>Participants: N =10 [6 males, 4 females]; mean (SD) age = 49.2 (11.8) years; duration of symptoms – median (range) = 12.9 (5-28) months; Numerical Pain Rating Scale (NRS) during activity – median (range) = 2.6 (1-6) | Qualitative, thematic analysis, interpretive description design using semi-structured interviews | Four themes identified:<br><br>1. Understanding the impact<br>2. Expectations<br>3. What matters<br>4. The burden of exercise |
| Mc Auliffe et al.[38]<br><br>Ireland | To explore the perceptions and experiences of people with Achilles tendinopathy (AT) prior to an intervention Study | Condition: Achilles tendinopathy<br><br>Participants: N = 8 [5 male, 3 female]; mean (SD) age – 40 (NR) years; duration of symptoms – median (range) – 20.5 (5-96) months; VISA-A | Qualitative, interpretive description design using semi-structured interviews | Four themes identified:<br><br>1. Pain as a feature of everyday life<br>2. Participants’ experience with management process<br>3. Identifying with and self-managing AT pain<br>4. Looking to the future. |

| | | | | |
|---|---|---|---|---|
| | | Score (/100) median (range) = 63.6 (28-81). | | |
| Nyman et al.[28]<br>Finland | To investigate the complexity of the effect of shoulder problems and discern and describe what it entails to be a patient suffering from shoulder problems | Condition: supraspinatus tendinitis<br>Participants: N = 21 [12 males, 9 females]; mean (SD) age = 53 (NR) years; duration of symptoms – mean (range) = NR (NR) | Qualitative design using focus group interviews | Four themes identified:<br>1. Sense of health condition (physical, mental)<br>2. Limitation (movement, other)<br>3. Control (own strategies, medication, information)<br>4. Expectations (positive, negative) |
| Palenius et al.[30]<br>Finland | To report how patients with shoulder problems experience physiotherapy practice in Finland. Do the patients have expectations of physiotherapists? How are the patients' experiences of physiotherapists? | Condition: Supraspinatus tendinitis<br>Participants: N = 26 [14 males, 12 females]; mean (SD) age= 53 (NR) years; duration of symptoms – median (range) = NR (NR) | Qualitative and phenomenological design using individual and focus group interviews | Eleven themes identified:<br>1. Examination<br>2. Manual therapy<br>3. Physical therapy<br>4. Program<br>5. Initial understanding<br>6. Communication<br>7. Motivating<br>8. Attention to wishes<br>9. Individualisation<br>10. Participation<br>11. Respect and dignity |
| Plinsinga et al.[40]<br>Australia | To explore participants' perspectives on, and experiences of, being assigned to a wait-and-see arm of a gluteal tendinopathy trial | Condition: Gluteal tendinopathy<br>Participants: N =15 [3 males, 12 females]; mean (SD) age = 56 (9); duration of symptoms – median (range) = 21 (8-144) months | Qualitative, inductive thematic approach using semi-structured interviews | Five themes were identified:<br>1. Feeling disenfranchised by being assigned to a wait-and-see approach<br>2. The importance of having a clinical and imaging diagnosis during screening for inclusion into the clinical trial<br>3. Feelings regarding the effectiveness of the approach<br>4. The convenient and easy to follow nature of the wait-and-see approach |

| | | | | |
|---|---|---|---|---|
| | | | | 5. The connotation of wait-and-see not always being perceived as an intervention |
| Powell et al.[35]<br>Australia | To gain insights into how individuals with rotator cuff-related shoulder pain believe exercise influenced their shoulder pain and identify the clinical conditions that promoted or inhibited their beliefs | Condition: Rotator cuff-related shoulder pain<br>Participants: N =11 [6 males, 5 females]; mean (SD) age = 36.5 (NR) years; duration of symptoms - median (range) = 29 (4-60) months | Qualitative, inductive thematic approach using semi-structured interviews | Six themes were identified:<br>1. A strong therapeutic relationship is the foundation for a clinical improvement with exercise therapy<br>2. The exercise program should be structured and tailored to the individual clinical presentation<br>3. Timely clinical progress with exercise therapy matters<br>4. Shoulder strength influences clinical outcomes<br>5. Exercise therapy influences psycho-emotional status<br>6. Exercise therapy has widespread positive health effects |
| Ryan et al.[39]<br>Ireland | To explore participants' experiences of partaking in a telehealth study including the acceptability of the intervention, motivators for participation, and perspectives on the trial processes | Condition: Achilles Tendinopathy<br>Participants: N = 16 [6 males, 10 females]; mean (SD) age = 45 (NR) years; duration of symptoms – median (range) = 11.9 (3-36) months | A qualitative follow-up study using semi-structured interviews undertaken on a group of participants from a previous pilot feasibility randomised controlled trial | Five themes identified:<br>1. The impact of Achilles Tendinopathy is commonly not prioritised, with 'The acceptance and minimisation of pain' as a sub-theme.<br>2. Therapeutic alliance has the greatest impact on support.<br>3. Factors which influenced adherence<br>4. Action Observation Therapy is valued and recommended.<br>5. Recommendations for future interventions. |
| Sole et al.[32]<br>New Zealand | To explore perceptions and initial outcomes of patients with rotator cuff- | Condition: Rotator cuff related pain<br>Participants: N =10 [5 males, 5 females]; median (SD) age = 57.5 (SD) years; duration of | Mixed-method design and general inductive approach using individual semi- | Two themes identified:<br>1. 'Participants' perspectives' of the session generated four themes (Improved understanding of 'the |

| | | | | |
|---|---|---|---|---|
| | related pain to a pain education session | symptoms – median (range) = 10 (4-24) months; SPADI pain median (range) = -13 (-40.0 to 10.0); SPADI function median (range) = -3.7 (-26.3 to 10.0); SPADI total median (range) = -9.2 (26.9 to 7.7); FABQ physical activity median (range) = -2.5 (-6 to 1); FABQ work median (range) = -3.5 (-10 to 1) | structured interviews and validated patient-reported outcomes measures. | whole'; Mindful self-awareness; Taking charge; "The pain is still there".<br>2. 'Participants' recommendations' had two themes: Integrating neuroscience with pathoanatomical knowledge and educating other health professionals. |
| Sandford et al.[31]<br>United Kingdom | To explore the barriers and enablers to adherence to a home- and class-based exercise program | Condition: Rotator cuff tendinopathy<br>Participants: N =12 [6 males, 6 females]; mean (SD) age = 54.8 (18.2) years; duration of symptoms – median (range) = 9.8 (NR) months; OSS median (range) = 14.17 (0-22) | Qualitative, inductive, and descriptive approach using semi-structured interviews | Three themes identified:<br>1. Experiences relating to participation in a scientific study<br>2. Self-efficacy<br>3. Enablers/facilitators and barriers to exercise |
| Stephens et al.[41]<br>United Kingdom | To provide insight into the experiences and perceptions of patients suffering with Greater Trochanteric Pain Syndrome (GTPS). | Condition: Greater Trochanteric Pain Syndrome<br>Participants: n =10 [1 male, 9 females]; mean (SD) age = 62.4 (NR) years; duration of symptoms – median (range) = 28.4 (4-72) months | Qualitative, inductive design using in depth semi-structured, telephone interviews | Five themes identified:<br>1. Living with persistent pain<br>2. Understanding the problem and pain<br>3. Experiences of previous treatment<br>4. Beliefs about activity and exercise<br>5. The future |
| Turner et al.[8]<br>United Kingdom | To explore the lived experiences of individuals with Achilles Tendinopathy (AT). | Condition: Achilles tendinopathy<br>Participants: N =15 [8 males, 7 females]; mean (SD) age = 45.2 (NR) years; duration of symptoms – median (range) =13.8 (3-96) months; VISA-A (/100) median (range) = 58.7 (19-85) | Qualitative, interpretive description design using semi-structured telephone interviews | Four themes identified:<br>1. Beliefs and perceptions surrounding Achilles tendinopathy<br>2. The biopsychosocial impact of Achilles tendinopathy<br>3. Individuals' experience with the management process<br>4. Future prognosis and outlook in individuals with Achilles tendinopathy |

| | | | | |
|---|---|---|---|---|
| Ulack et al.[33]<br>USA | To explore the daily experience of living with rotator cuff tendinopathy (RCT). | Condition: Rotator cuff tendinopathy<br>Participants: N =19 [9 males, 10 females]; mean (SD) age = 54 (NR) years; duration of symptoms – median (range) = NR (NR) | Qualitative, inductive thematic approach using semi-structured group sessions | Three domains with themes identified:<br>1. Capability:<br>a. Restoring regular sleeping patterns<br>b. Finding suitable work<br>c. Regaining identity (both at home and at work)<br>d. Engaging in meaningful activity with loved ones<br>2. Comfort:<br>a. Restoring mental health (depression)<br>b. Reducing pain associated with treatment<br>3. Calm:<br>a. Building a relationship that leads you to success<br>b. Depression<br>c. Therapy itself sometimes exacerbates pain<br>d. Perception of condition as an injury rather than chronic disease<br>e. Lack of clarity in diagnosis led to uncertainty/confusion about the disease<br>f. Strained patient-clinician relationship<br>g. Patients sometimes “manipulate” MDs to get the care they need<br>h. Patients do not always trust that doctors are providing necessary care<br>i. Pain medications impede on work and social life<br>j. Financial burden of disease |

NB: NR: not reported, rESWT: radial extracorporeal shockwave therapy, SD: standard deviation, VISA-A Score: Victorian Institute of Sport Assessment-Achilles Questionnaire, AQoL-8D: 8-dimension Assessment of Quality-of-Life, RCRSP: rotator cuff related shoulder pain, RCT: Randomised controlled trial , GTPS: Greater Trochanteric Pain Syndrome, AT: Achilles Tendinopathy, RCT: Rotator cuff tendinopathy , MDs: Medical Doctors, SPADI: The Shoulder Pain and Disability Index, PSEQ: The Pain Self-Efficacy Questionnaire, PRTEE: Patient Reported Tennis Elbow Evaluation, NRS: Numerical Pain Rating Scale, FABQ: Fear Avoidance Beliefs Questionnaire, OSS: Oxford Shoulder Score

**Supplementary Material 4.** Grading of Recommendations Assessment, Development and Evaluation Confidence in the Evidence from Reviews of Qualitative research (GRADE-CERQual) evidence profile

| **Summary of review finding** | **Studies contributing to the review finding** | **Methodological limitations** | **Coherence** | **Adequacy** | **Relevance** | **CERQual assessment of confidence in the evidence** | **Explanation of CERQual assessment** |
|---|---|---|---|---|---|---|---|
| 1. Patient education and knowledge about tendinopathy: Participants with tendinopathy desired to understand why their tendon hurts seeking clarity in what they were dealing with whether from online resources or healthcare professionals. | [7,9,25-37,39,40,42] | Moderate Methodological limitations<br><br>(12 studies with minor and 6 studies with moderate methodological limitations (Unclear method of sampling and recruitment causing bias in participants' views,[25,29,32,42] short length of interviews approx.13 mins leading to superficial answers from participants,[26] data collection and analysis by one individual which may hamper transferability, credibility and | No or very minor concerns | Minor concerns about adequacy<br><br>(17 studies that together offering moderately rich data and one study offering thin data[26]) | Minor concerns about relevance<br><br>(All studies included individuals with tendinopathy across 5 different high-income countries within a wide range of clinical settings) | Moderate confidence | Moderate concerns regarding methodological limitations, and minor concerns regarding coherence, adequacy and relevance of data. |

| | | | | | | | |
|---|---|---|---|---|---|---|---|
| | | confirmability of the findings,[27] and iterative approach leading to risk of researcher bias and potential narrowing of the topic field[31])) | | | | | |
| 2. Patient experience with management of tendinopathy: Participants with tendinopathy desired to treat their tendon pain. However, they are not sure what ’ s the best method to do that; whether it’ s exercises or passive treatment (e.g., massages) or injection therapy or just rest and do nothing. | 8,9,18,26-30,34,35,38,39,41,42 | Moderate Methodological limitations<br><br>(10 studies with minor and 4 studies with moderate methodological limitations (short length of interviews approx.13 mins leading to superficial answers from participants,[26] data collection and analysis by one individual which may hamper transferability, credibility and confirmability of the findings,[27] use of pre-determined themes and mostly deductive approach to | No or very minor concerns | Minor concerns about adequacy<br><br>(11 studies that together offering moderately rich data and 3 studies offering thin data[18,26,41]) | Minor concerns about relevance<br><br>(All studies included individuals with tendinopathy across 5 different high-income countries within a wide range of clinical settings) | Moderate confidence | Moderate concerns regarding methodological limitations, and minor concerns regarding coherence, adequacy and relevance of data. |

| | | | | | | | |
|---|---|---|---|---|---|---|---|
| | | data analysis could mean that that some emerging themes may have been missed,[41] unclear recruitment and sampling from one private clinic[42])) | | | | | |
| 3. Impact of tendinopathy on patient lifestyle: Participants with tendinopathy expressed that they were uncertain whether their lifestyle will return to normal with regards to their sports, jobs or even social activities with their friends and families. | [6-9,18,28,29,33,34,37,38] | Minor methodological limitations<br>(All studies had minor methodological limitations (no reflexivity)) | No or very minor concerns | Minor concerns about adequacy<br>(9 studies that together offering moderately rich data and 2 studies offering thin data[6,18]) | Minor concerns about relevance<br>(All studies included individuals with tendinopathy across 7 different high-income countries within a wide range of clinical settings) | High confidence | Minor concerns regarding methodological limitations, coherence, adequacy and relevance of data. |

**NB: The GRADE-CERQual confidence ratings are determined through systematic assessment of four key components: (1) Methodological limitations: evaluating the quality of primary studies contributing to a review finding; (2) Coherence: assessing the fit between data from primary studies and the synthesised review finding; (3) Adequacy of data: evaluating the richness and quantity of data supporting a review finding; and (4) Relevance: determining the applicability of primary study evidence to the context specified in the review question. Authors assess each component individually, identifying concerns that could reduce confidence in the review finding, judging whether there are no/very minor, minor, moderate, or serious concerns. An overall confidence assessment starts from high and may be downgraded based on concerns in each component. Final ratings (high, moderate, low, or very low) reflect the extent to which the review finding reasonably represents the phenomenon of interest.[83]**

# Reference List


1. Scott, Alex, Backman LJ, Speed C. Tendinopathy: update on pathophysiology. *The Journal of Orthopaedic and Sports Physical Therapy*. 2015;45(11):833-841. doi:10.2519/jospt.2015.5884
2. Millar NL, Silbernagel KG, Thorborg K, et al. Tendinopathy. *Nature Reviews Disease Primers*. 2021;7(1):1-1. doi:10.1038/s41572-020-00234-1
3. Sleeswijk Visser TSO, van der Vlist AC, van Oosterom RF, van Veldhoven P, Verhaar JAN, de Vos R-J. Impact of chronic Achilles tendinopathy on health-related quality of life, work performance, healthcare utilisation and costs. *British Medical Journal Open Sport & Exercise Medicine*. 2021;7(1):e001023-e001023. doi:10.1136/bmjsem-2020-001023
4. Silbernagel KG, Hanlon S, Sprague A. Current clinical concepts: conservative management of Achilles tendinopathy. *Journal of Athletic Training*. 2020;55(5):438-447. doi:10.4085/1062-6050-356-19
5. Malliaras P, Johnston R, Street G, et al. The efficacy of higher versus lower dose exercise in rotator cuff tendinopathy: a systematic review of randomized controlled trials. *Archives of Physical Medicine and Rehabilitation*. 2020;101(10):1822-1834. doi:10.1016/j.apmr.2020.06.013
6. Lee, Lee MJ. A study on tennis elbow patient's experiences of alternative therapy use in Korea. *International Journal of Bio-Science and Bio-Technology*. 2016;8(2):121-132. doi:http://dx.doi.org/10.14257/ijbsbt.2016.8.2.11
7. Bateman M, Hill JC, Cooper K, Littlewood C, Saunders B. Lived experience of people with lateral elbow tendinopathy: a qualitative study from the OPTimisE pilot and feasibility trial. *British Medical Journal Open*. 2023;13(8):e072070-e072070. doi:10.1136/bmjopen-2023-072070
8. Turner J, Malliaras P, Goulis J, Mc Auliffe S. "It's disappointing and it's pretty frustrating, because it feels like it's something that will never go away." a qualitative study exploring individuals' beliefs and experiences of Achilles tendinopathy. *Public Library of Science One*. 2020;15(5):e0233459-e0233459. doi:10.1371/journal.pone.0233459
9. Gillespie MAB, Mącznik AMP, Wassinger CAPTP, Sole GBMSP. Rotator cuff-related pain: Patients' understanding and experiences. *Musculoskeletal Science and Practice*. 2017;30:64-71. doi:10.1016/j.msksp.2017.05.009
10. Childress MAMD, Beutler AMD. Management of chronic tendon injuries. *American Family Physician*. 2013;87(7):486-490.
11. Liberati A, Altman DG, Tetzlaff J, et al. The PRISMA statement for reporting systematic reviews and meta-analyses of studies that evaluate health care interventions: explanation and elaboration. *The Public Library of Science Medicine* 2009;6(7):e1000100-e1000100. doi:10.1371/journal.pmed.1000100
12. France, Cunningham M, Ring N, et al. Improving reporting of meta-ethnography: the eMERGe reporting guidance. *Journal of Advanced Nursing*. 2019;75(5):1126-1139. doi:10.1111/jan.13809
13. France, Ring N, Noyes J, et al. Protocol-developing meta-ethnography reporting guidelines (eMERGe). *BMC Medical Research Methodology*. 2015;15(1):103-103. doi:10.1186/s12874-015-0068-0

14. Sattar RL, Rebecca ; Panagioti, Maria ; Johnson, Judith. Meta-ethnography in healthcare research: a guide to using a meta-ethnographic approach for literature synthesis. *BioMed Central Health Services Research* 2021;21:50-50.
15. France EF, Wells M, Lang H, Williams B. Why, when and how to update a meta-ethnography qualitative synthesis. *Systematic reviews*. 2016;5(1):44-44. doi:10.1186/s13643-016-0218-4
16. Toye F, Seers K, Hannink E, Barker K. A mega-ethnography of eleven qualitative evidence syntheses exploring the experience of living with chronic non-malignant pain. *BMC Medical Research Methodology*. 2017;17(1):116-116. doi:10.1186/s12874-017-0392-7
17. Scott, Squier K, Alfredson H, et al. ICON 2019: international scientific tendinopathy symposium consensus: clinical terminology. Article. *British Journal of Sports Medicine*. 2020;54(5):260-262. doi:10.1136/bjsports-2019-100885
18. Ceravolo ML, Gaida JE, Keegan RJ. Quality-of-life in achilles tendinopathy: an exploratory study. *Clinical Journal of Sport Medicine*. 2020;30(5):495-502. doi:10.1097/JSM.0000000000000636
19. Briggs J. Checklist for qualitative research https://jbi.global/sites/default/files/2019-05/JBI_Critical_Appraisal-Checklist_for_Qualitative_Research2017_0.pdf
20. Cohen. A coefficient of agreement for nominal scales. *Educational and Psychological Measuremen*. 1960;20:37-46.
21. Noblit, Hare RD. *Meta-ethnography: synthesising qualitative studies*. 11 ed. London: Sage Publications; 1988.
22. NVivo L. Leading Qualitative Data Analysis Software. https://lumivero.com/products/nvivo/?utm_source=google&utm_medium=search_paid&utm_campaign=nv_ROW_go_acq_leadgen_brand&utm_content=nv_ROW_go_acq_leadgen_brand_nvivo_exact&utm_ad=688061603403&utm_term=nvivo%2012%20software&matchtype=e&device=c&GeoLoc=9071728&placement=&network=g&campaign_id=20397585540&adset_id=156520034543&ad_id=688061603403&gad_source=1&gclid=EAIaIQobChMIybGe4573gwMV0sY8Ah3tww-pEAAYAiAAEgLZ2_D_BwE
23. Campbell R, Pound P, Morgan M, et al. Evaluating meta-ethnography: systematic analysis and synthesis of qualitative research. Review. *Health Technology Assessment*. 2011;15(43):i-164. doi:10.3310/hta15430
24. Lewin S, Glenton C, Munthe-Kaas H, et al. Using qualitative evidence in decision making for health and social interventions: an approach to assess confidence in findings from qualitative evidence syntheses (GRADE-CERQual). *PLoS medicine*. 2015;12(10):e1001895-e1001895. doi:10.1371/journal.pmed.1001895
25. Cridland K, Pritchard S, Rathi S, Malliaras P. 'He explains it in a way that I have confidence he knows what he is doing': A qualitative study of patients' experiences and perspectives of rotator-cuff-related shoulder pain education. *Musculoskeletal Care*. 2021;19(2):217-231. doi:10.1002/msc.1528
26. Kiely D. *Group versus individual treatment in the management of rotator cuff tendinopathy in primary care.* University of Limerick; 2021.
27. Littlewood C, Malliaras P, Mawson S, May S, Walters S. Patients with rotator cuff tendinopathy can successfully self-manage, but with certain caveats: a qualitative study. *Physiotherapy*. 2013;100(1):80-85. doi:10.1016/j.physio.2013.08.003
28. Nyman P, Palenius K, Panula H, Mälkiä PPTE. Patients' experiences of shoulder problems prior to and following intervention. *Physiotherapy Theory and Practice*. 2012;28(3):221-231. doi:10.3109/09593985.2011.598220
29. Malliaras P, Rathi S, Burstein F, et al. 'Physio's not going to repair a torn tendon': patient decision-making related to surgery for rotator cuff related shoulder pain. *Disability and Rehabilitation*. 2021:1-8. doi:10.1080/09638288.2021.1879945

30. Palenius KG, Nyman PC. Expectations and experiences of physiotherapeutic practice among patients with shoulder problems. *European Journal of Physiotherapy*. 2018;20(1):58-64. doi:10.1080/21679169.2017.1363283
31. Sandford FMPM, Sanders TABDPRN, Lewis JSPFM. Exploring experiences, barriers, and enablers to home- and class-based exercise in rotator cuff tendinopathy: a qualitative study. *Journal of Hand Therapy*. 2017;30(2):193-199. doi:10.1016/j.jht.2017.05.001
32. Sole G, Mącznik AK, Ribeiro DC, Jayakaran P, Wassinger CA. Perspectives of participants with rotator cuff-related pain to a neuroscience-informed pain education session: an exploratory mixed method study. *Disability and Rehabilitation*. 2020;42(13):1870-1879. doi:10.1080/09638288.2018.1542037
33. Ulack C, Suarez J, Brown L, Ring D, Wallace S, Teisberg E. What are people that seek care for rotator cuff tendinopathy experiencing in their daily life? *Journal of Patient Experience*. 2022;9:23743735211069811-23743735211069811. doi:10.1177/23743735211069811
34. Acker R, Swain N, Perry M, Wassinger C, Sole G. 'Thinking about pain in a different way': Patient perspectives of a neuroscience-informed physiotherapy programme for rotator cuff-related shoulder pain. Article. *Musculoskeletal Science and Practice*. 2023;63doi:10.1016/j.msksp.2022.102691
35. Powell JK, Costa N, Schram B, Hing W, Lewis J. "Restoring that faith in my shoulder": A qaualitative investigation of how and why exercise therapy influenced the clinical outcomes of individuals with rotator cuff-related shoulder pain. *Physical Therapy*. 2023;doi:10.1093/ptj/pzad088
36. Hasani F, Malliaras P, Haines T, et al. Telehealth sounds a bit challenging, but it has potential: participant and physiotherapist experiences of gym-based exercise intervention for Achilles tendinopathy monitored via telehealth. *BMC Musculoskeletal Disorders*. 2021;22(1):138-138. doi:10.1186/s12891-020-03907-w
37. Mallows A, Head J, Goom T, Malliaras P, O'Neill S, Smith B. Patient perspectives on participation in exercise-based rehabilitation for Achilles tendinopathy: a qualitative study. *Musculoskeletal Science and Practice*. 2021;56:102450-102450. doi:10.1016/j.msksp.2021.102450
38. Mc Auliffe S, Synott A, Casey H, Mc Creesh K, Purtill H, O'Sullivan K. Beyond the tendon: experiences and perceptions of people with persistent Achilles tendinopathy. *Musculoskeletal Science & Practice*. 2017;29:108-114. doi:10.1016/j.msksp.2017.03.009
39. Ryan D, Rio E, O'Donoghue G, O'Sullivan C. "I've got a spring in my step" participants experience of action observation therapy and eccentric exercises, a telehealth study for mid-portion Achilles Tendinopathy: a qualitative study. Article. *Journal of foot and ankle research*. 2023;16(1):19. doi:10.1186/s13047-023-00619-x
40. Plinsinga ML, Mellor R, Setchell J, et al. Perspectives and experiences of people who were randomly assigned to wait-and-see approach in a gluteal tendinopathy trial: a qualitative follow-up study. *British Medical Journal Open*. 2021;11(4):e044934-e044934. doi:10.1136/bmjopen-2020-044934
41. Stephens G, O'Neill S, Mottershead C, Hawthorn C, Yeowell G, Littlewood C. "It's just like a needle going into my hip, basically all of the time". The experiences and perceptions of patients with greater trochanteric pain syndrome in the UK national health service. *Musculoskelet Science Practice*. 2020;47:102175-102175. doi:10.1016/j.msksp.2020.102175
42. Leung R, Malliaropoulos N, Korakakis V, Padhiar N. What are patients' knowledge, expectation and experience of radial extracorporeal shockwave therapy for the treatment of their tendinopathies? a qualitative study. *Journal of Foot and Ankle Research*. 2018;11(1):11-11. doi:10.1186/s13047-018-0254-5

43. Mobini S. Psychology of medically unexplained symptoms: A practical review. *Cogent Psychology*. 2015;2(1):1033876. doi:10.1080/23311908.2015.1033876
44. Sowińska A, Czachowski S. Patients' experiences of living with medically unexplained symptoms (MUS): a qualitative study. *BioMed Central Family Practice*. 2018;19(1):23-23. doi:10.1186/s12875-018-0709-6
45. Wuytack F, Miller P. The lived experience of fibromyalgia in female patients, a phenomenological study. *Chiropractic & manual therapies*. 2011;19(1):22-22. doi:10.1186/2045-709X-19-22
46. Dima A, Lewith GT, Little P, Moss-Morris R, Foster NE, Bishop FL. Identifying patients' beliefs about treatments for chronic low back pain in primary care: a focus group study. *British Journal of General Practice*. 2013;63(612):e490-e498. doi:10.3399/bjgp13X669211
47. Erwin J, Chance-Larsen K, Backhouse M, Woolf AD. Exploring what patients with musculoskeletal conditions want from first point-of-contact health practitioners. *Rheumatology Advances in Practice*. 2020;4(1):rkz048-rkz048. doi:10.1093/rap/rkz048
48. Majid N, Lee S, Plummer V. The effectiveness of orthopedic patient education in improving patient outcomes: a systematic review protocol. *JBI Database of Systematic Reviews and Implementation Reports*. 2015;13(1):122-133. doi:10.11124/jbisrir-2015-1950
49. Lieberthal K, Paterson KL, Cook J, Kiss Z, Girdwood M, Bradshaw EJ. Prevalence and factors associated with asymptomatic Achilles tendon pathology in male distance runners. *Physical Therapy in Sport*. 2019;39:64-68. doi:10.1016/j.ptsp.2019.06.006
50. White J, Auliffe SM, Jepson M, et al. 'There is a very distinct need for education' among people with rotator cuff tendinopathy: An exploration of health professionals' attitudes. *Musculoskeletal Science & Practice*. 2020;45:102103-102103. doi:10.1016/j.msksp.2019.102103
51. Krist AH, Tong ST, Aycock RA, Longo DR. Engaging patients in decision-making and behavior change to promote prevention. *Information Services & Use*. 2017;37(2):105-122. doi:10.3233/ISU-170826
52. Leonard P. Exploring ways to manage healthcare professional—patient communication issues. *Supportive Care In Cancer*. 2017;25(Suppl 1):7-9. doi:10.1007/s00520-017-3635-6
53. Desjardins-Charbonneau A, Roy J-S, Dionne CE, Frémont P, MacDermid JC, Desmeules F. The efficacy of manual therapy for rotator cuff tendinopathy: a systematic review and meta-analysis. *The Journal of Orthopaedic and Sports Physical Therapy*. 2015;45(5):330-350. doi:10.2519/jospt.2015.5455
54. Stefansson SH, Brandsson S, Langberg H, Arnason A. Using pressure massage for Achilles tendinopathy: A single-blind, randomized controlled trial comparing a novel treatment versus an eccentric exercise protocol. *Orthopaedic Journal of Sports Medicine*. 2019;7(3):2325967119834284-2325967119834284. doi:10.1177/2325967119834284
55. Challoumas D, Crosbie G, O'Neill S, Pedret C, Millar NL. Effectiveness of exercise treatments with or without adjuncts for common lower limb tendinopathies: A living systematic review and network meta-analysis. *Sports Medicine - Open*. 2023;9(1):71-14. doi:10.1186/s40798-023-00616-1
56. Ortega-Castillo M, Cuesta-Vargas A, Luque-Teba A, Trinidad-Fernández M. The role of progressive, therapeutic exercise in the management of upper limb tendinopathies: A systematic review and meta-analysis. Review. *Musculoskeletal Science and Practice*. 2022;62doi:10.1016/j.msksp.2022.102645

57. Karanasios S, Korakakis V, Whiteley R, Vasilogeorgis I, Woodbridge S, Gioftsos G. Exercise interventions in lateral elbow tendinopathy have better outcomes than passive interventions, but the effects are small: a systematic review and meta-analysis of 2123 subjects in 30 trials. *British Journal of Sports Medicine*. 2021;55(9):477-485. doi:10.1136/bjsports-2020-102525
58. Constand MK, MacDermid JC, Dal Bello-Haas V, Law M. Scoping review of patient-centered care approaches in healthcare. *BioMed Central Health Services Research*. 2014;14(1):271-271. doi:10.1186/1472-6963-14-271
59. Greene SM, Tuzzio L, Cherkin D. A framework for making patient-centered care front and center. *Permanente Journal*. 2012;16(3):49-53. doi:10.7812/tpp/12-025
60. Marks D, Window P, Raymer M, et al. Exploring Congruence Between Patient and Clinician Expectations of Benefit in the Non-Surgical Management of Common Musculoskeletal Conditions in Tertiary Care. *Musculoskeletal care*. 2024;22(4):e70036-n/a. doi:10.1002/msc.70036
61. Bialosky JE, Bishop MD, Cleland JA. Individual expectation: an overlooked, but pertinent, factor in the treatment of individuals experiencing musculoskeletal pain. *Physical Therapy*. 2010;90(9):1345-1355. doi:10.2522/ptj.20090306
62. Teo JL, Zheng Z, Bird SR. Identifying the factors affecting 'patient engagement' in exercise rehabilitation. *BioMed Central Sports Science, Medicine and Rehabilitation*. 2022;14(1):18-18. doi:10.1186/s13102-022-00407-3
63. Wallis JA, Taylor NF, Bunzli S, Shields N. Experience of living with knee osteoarthritis: a systematic review of qualitative studies. *British Medical Journal Open*. 2019;9(9):e030060-e030060. doi:10.1136/bmjopen-2019-030060
64. MacNeela P, Doyle C, O'Gorman D, Ruane N, McGuire BE. Experiences of chronic low back pain: a meta-ethnography of qualitative research. *Health Psychology Review*. 2015;9(1):63-82. doi:10.1080/17437199.2013.840951
65. Kwame A, Petrucka PM. A literature-based study of patient-centered care and communication in nurse-patient interactions: barriers, facilitators, and the way forward. *BioMed Central Nursing*. 2021;20(1):1-10. doi:10.1186/s12912-021-00684-2
66. Santana M-J, Manalili K, Zelinsky S, et al. Improving the quality of person-centred healthcare from the patient perspective: development of person-centred quality indicators. *British Medical Journal Open*. 2020;10(10):e037323. doi:10.1136/bmjopen-2020-037323
67. Adams RJ. Improving health outcomes with better patient understanding and education. *Risk Management and Healthcare Policy*. 2010;3(default):61-72. doi:10.2147/RMHP.S7500
68. Mallows A, Debenham J, Walker T, Littlewood C. Association of psychological variables and outcome in tendinopathy: a systematic review. *British Journal of Sports Medicine*. 2017;51(9):743-748. doi:10.1136/bjsports-2016-096154
69. Hashim MJMD. Patient-centered communication: basic skills. *American Family Physician*. 2017;95(1):29-34.
70. Sharkiya SH. Quality communication can improve patient-centred health outcomes among older patients: a rapid review. *BioMedical Central health services research*. 2023;23(1):1-886. doi:10.1186/s12913-023-09869-8
71. King A, Hoppe RB. "Best practice" for patient-centered communication: a narrative review. *Journal of Graduate Medical Education*. 2013;5(3):385-393. doi:10.4300/jgme-d-13-00072.1
72. Su M, Zhou Z, Si Y, Fan X. The association between patient-centered communication and primary care quality in urban China: evidence from a standardized patient Sstudy. *Frontiers in Public Health*. 2022;9:779293-779293. doi:10.3389/fpubh.2021.779293
73. Malliaras P, Cook J, Purdam C, Rio E. Patellar tendinopathy: clinical diagnosis, load management, and advice for challenging case presentations. *The Journal of Orthopaedic and Sports Physical Therapy*. 2015;45(11):887-898. doi:10.2519/jospt.2015.5987

74. Australia A. Understand & manage your back pain. https://mybackpain.org.au/
75. Talevski J, Wong Shee A, Rasmussen B, Kemp G, Beauchamp A, Mathes T. Teach-back: A systematic review of implementation and impacts. *Nonprofit Open-Access Publisher One*. 2020;15(4):e0231350-e0231350. doi:10.1371/journal.pone.0231350
76. Hawkins M, Elsworth GR, Osborne RH. Questionnaire validation practice: a protocol for a systematic descriptive literature review of health literacy assessments. *BMJ open*. 2019;9(10):e030753-e030753. doi:10.1136/bmjopen-2019-030753
77. Darnall BD, Sturgeon JA, Cook KF, et al. Development and validation of a daily pain catastrophizing scale. *The Journal of Pain*. 2017;18(9):1139-1149. doi:10.1016/j.jpain.2017.05.003
78. Aisami RS. Learning styles and visual literacy for learning and performance. *Procedia, Social and Behavioral Sciences*. 2015;176:538-545. doi:10.1016/j.sbspro.2015.01.508
79. Clifford C, Challoumas D, Paul L, Syme G, Millar NL. Effectiveness of isometric exercise in the management of tendinopathy: a systematic review and meta-analysis of randomised trials. *British Medical Journal Open Sport & Exercise Medicine*. 2020;6(1):e000760-e000760. doi:10.1136/bmjsem-2020-000760
80. Derksen F, Bensing J, Lagro-Janssen A. Effectiveness of empathy in general practice: a systematic review. *British Journal of General Practice*. 2013;63(606):e76-e84. doi:10.3399/bjgp13x660814
81. Marcinowicz L, Konstantynowicz J, Godlewski C. Patients' perceptions of GP non-verbal communication: a qualitative study. *British Journal of General Practice*. 2010;60(571):83-87. doi:10.3399/bjgp10x483111
82. Littlewood C, Bateman M, Brown K, et al. A self-managed single exercise programme versus usual physiotherapy treatment for rotator cuff tendinopathy: a randomised controlled trial (the SELF study). *Clinical Rehabilitation*. 2016;30(7):686-696. doi:10.1177/0269215515593784
83. Lewin S, Bohren M, Rashidian A, et al. Applying GRADE-CERQual to qualitative evidence synthesis findings-paper 2: how to make an overall CERQual assessment of confidence and create a summary of qualitative findings table. *Implementation Science* 2018;13(Suppl 1):10-10. doi:10.1186/s13012-017-0689-2
